# A cis-eQTL of HLA-DPB1 Affects Susceptibility to Type 1 Autoimmune Hepatitis

**DOI:** 10.1038/s41598-018-30406-9

**Published:** 2018-08-09

**Authors:** Tomoo Yamazaki, Takeji Umemura, Satoru Joshita, Kaname Yoshizawa, Eiji Tanaka, Masao Ota

**Affiliations:** 1 Department of Medicine, Division of Hepatology and Gastroenterology, Shinshu University School of Medicine, Matsumoto, Japan; 2 Research Center for Next Generation Medicine, Shinshu University, Matsumoto, Japan; 3 Department of Gastroenterology, NHO Ueda Medical Center, Ueda, Japan

## Abstract

Autoimmune hepatitis (AIH) is a chronic inflammatory liver disease characterized by an autoimmune reaction to hepatocytes. A single nucleotide polymorphism in the 3′ untranslated region of HLA-DPB1, rs9277534, is associated with HLA-DPB1 expression. rs9277534 has been linked to hepatitis B virus recovery/persistence and the risk of graft-versus-host disease with HLA-DPB1 mismatching transplantation of hematopoietic cells, but its role along with that of HLA-DP expression in AIH have not been fully clarified. We genotyped rs9277534 in 146 Japanese patients with AIH and 326 healthy subjects. HLA-DPB1 expression was determined by quantitative PCR. HLA-DPB1 expression was significantly higher for rs9277534G than for rs9277534A (*P* < 0.05). rs9277534 genotype was in strong linkage disequilibrium with the HLA-DPB1 allele (pairwise D′ = 0.82–1.00). Although HLA-DP alleles were not significantly associated with AIH, the frequency of the rs9277534G allele was significantly higher in AIH patients compared with healthy subjects (*P* = 0.002, odds ratio [OR] = 1.56). Logistic regression analysis revealed that the HLA-DRB1*04:05 allele (*P* < 0.001, OR = 4.61) and rs9277534 (*P* = 0.004, OR = 1.67) were independently associated with AIH susceptibility. rs9277534G in the HLA-DP gene is an eQTL that affects gene expression and may contribute to AIH susceptibility.

## Introduction

Autoimmune hepatitis (AIH) is a chronic inflammatory disease of the liver characterized by elevated transaminase, hypergammaglobulinemia, the presence of serum autoantibody, and necroinflammatory changes on hepatic histology^1,2^. Two types of AIH have been defined by serum autoantibody profiles^3,4^. Type 1 AIH is characterized by the presence of antinuclear antibodies and/or anti-smooth muscle antibodies and is the major form of AIH in Japan. Although the disease is believed to result from a combination of genetic and environmental factors, its exact etiology remains unclear. Many studies have implicated human leukocyte antigen (HLA) class II genes, especially *HLA-DRB1* alleles, as major components in the genetic predisposition to AIH. For instance, the DRB1*04:04 and *DRB1:04:05* alleles are linked to susceptibility in Mexican^5^ and Japanese^6–8^ and Korean^9^ populations, respectively, and studies including recent genome-wide association analyses have shown that DRB1*03:01 and DRB1*04:01 contribute to AIH susceptibility in European populations^10–12^.

*HLA-DP* genes have not been examined for their impact on human disease relative to HLA-*DR* and -*DQ* because they are less polymorphic and HLA-DP cell surface expression levels are believed to be lower than those of HLA-DR or -DQ^13–15^. Several genome-wide association studies have associated *HLA-DP* gene variations with chronic hepatitis B virus (HBV) infection in Asians^16–20^. Moreover, two single nucleotide polymorphisms (SNPs) in the 3′ untranslated region of HLA-DPB1, rs9277534 and rs9277535, are related to viral clearance in the U.S. population^21^ and in Asians^16^, respectively. Petersdorf *et al*. recently found that the risk of graft-versus-host disease with HLA-DPB1 mismatching transplantation of hematopoietic cells was influenced by rs9277534^22^. Other current findings^21,22^ suggest that this SNP is associated with HLA-DP expression levels and may influence the strength of autoimmune responses, thus representing a new and crucial role of HLA in human disease. We therefore sought to determine whether rs9277534 and HLA-DP expression were associated with AIH in a Japanese population.

## Results

### HLA-DPB1 Alleles and rs9277534 in AIH Patients and Controls

The frequency of the major G allele in rs9277534 was significantly increased in AIH patients as compared with 326 healthy subjects (65.1% vs. 54.3%, *P = *0.002, odds ratio [OR] = 1.56) (Table 1). Genotype frequencies also differed significantly between AIH patients and controls with a dominant model of inheritance (Table 2). The frequency of the GG or GA genotype of rs9277534 in AIH was significantly higher than that in controls (*P* = 0.0034, OR = 2.39).

**Table 1:** Allelic Association Tests of rs9277534 in Patients with AIH.

SNP	Position	Gene location	Alleles (1 > 2)	Major allele frequency, %		Controls vs. AIH	
				Controls (n = 326)	AIH (n = 146)	*P* value	OR (95% CI)
rs9277534 (HLA-DPB1)	Chr. 6 33087030	3′UTR	G > A	54.3	65.1	0.002	1.56 (1.18–2.09)

Abbreviations: AIH, autoimmune hepatitis; 1, major allele; 2, minor allele; OR, odds ratio; CI, confidence interval.

**Table 2:** Genotype Distribution of HLA Gene Polymorphisms in Patients with AIH and Healthy Subjects.

SNP	Alleles (1 > 2)	Genotypes	Genotype frequency, %		Model	Controls vs. AIH	
			Controls (n = 326)	AIH (n = 146)		*P* value	OR (95% CI)
rs9277534 (HLA-DPB1)	G > A	GG/GA/AA	30.1/48.5/21.5	40.4/49.3/10.3	GG/GA + AA	0.027	1.58 (1.05–2.37)
					GG + GA/AA	0.0034	2.39 (1.32–4.33)

*The model with the smallest Akaike’s information criterion value was defined as the most appropriate for each SNP.

Abbreviations: HLA, human leukocyte antigen; AIH, autoimmune hepatitis; 1, major allele; 2, minor allele; OR, odds ratio; CI, confidence interval; SNP, single nucleotide polymorphism.

*HLA-DP* allele frequency between 146 patients with AIH and 201 control subjects from our prior study^23^ was compared next (Table 3). DPB1*04:02 had a lower frequency in patients, but this difference was not significant after correction for multiple testing (8% vs. 13%; *P* = 0.023, corrected *P = *0.28). In our cohort, the HLA-DRB1*04:05 allele is strongly associated with AIH susceptibility (*P* < 0.001), as is rs9277534 (*P* = 0.004). Logistic regression analysis revealed that the HLA-DRB1*04:05 allele (*P* < 0.001, OR = 4.61, 95% CI = 2.86–7.41) and rs9277534 (*P* = 0.004, OR = 1.67, 95% CI = 1.18–2.37) were independently associated with AIH susceptibility.

**Table 3:** Statistical Analysis of Representative HLA-DPB1 Alleles in Patients with AIH and Healthy Subjects.

Allele	Frequency, n (%)		*P* value	OR (95% CI)
	Patients (2n = 292)	Controls (2n = 402)		
DPB1*02:01	58 (20)	98 (24)	0.16	
DPB1*02:02	7 (2)	16 (4)	0.25	
DPB1*03:01	16 (5)	13 (3)	0.14	
DPB1*04:01	11 (4)	24 (6)	0.19	
DPB1*04:02	22 (8)	52 (13)	0.023^†^	0.55 (0.33–0.92)
DPB1*05:01	132 (45)	156 (39)	0.091	
DPB1*06:01	1 (0)	0 (0)	0.24	
DPB1*09:01	29 (10)	29 (7)	0.20	
DPB1*13:01	2 (1)	7 (2)	0.22	
DPB1*14:01	8 (3)	5 (1)	0.15	
DPB1*17:01	1 (0)	0 (0)	0.24	
DPB1*19:01	5 (2)	2 (0)	0.11	

^†^Corrected *P* = 0.28.

Abbreviations: AIH, autoimmune hepatitis; OR, odds ratio; CI, confidence interval.

### Association between HLA-DPB1 and rs9277534 Allele Type

The ratio of GG/GA/AA genotypes in rs9277534 was 0.301/0.485/0.215 and the respective allele frequency of G and A was 0.543 and 0.457 in 326 healthy subjects (Tables 1, 2). These were in deviation of Hardy-Weinberg equilibrium (*P* = 0.914). We carried out *HLA-DPB1* allele typing in 146 AIH patients and analyzed for associations between *HLA-DPB1* and rs9277534 allele type (Table 4). HLA-DPB1*02:01, DPB1*02:02, DPB1*04:01, DPB1*04:02, and DPB1*17:01 alleles were associated with rs9277534A, while HLA-DPB1*03:01, DPB1*05:01, DPB1*06:01, DPB1*09:01, DPB1*13:01, DPB1*14:01, and DPB1*19:01 were linked with rs9277534G. The genotype of rs9277534 was in strong linkage disequilibrium with the *HLA-DPB1* allele (pairwise *D*′ = 0.82–1.00). The *HLA-DPB1* alleles could be divided into two groups by the rs9277534 allele, which was consistent with a recent report^21^.

**Table 4:** Linkage Disequilibrium between HLA-DPB1 Allele Type and rs9277534 in Patients with AIH.

rs9277534	Allele	Observed frequency	*D*′	*r* ^2^	*P* value
A	*DPB*1*02:01	0.199	1.00	0.47	<0.001
A	*DPB*1*02:02	0.024	1.00	0.05	<0.001
A	DPB1*04:01	0.038	0.86	0.05	<0.001
A	*DPB*1*04:02	0.075	1.00	0.15	<0.001
A	DPB1*17:01	0.003	1.00	0.00	0.46
G	DPB1*03:01	0.055	0.82	0.02	0.01
G	*DPB*1*05:01	0.452	0.93	0.38	<0.001
G	DPB1*06:01	0.003	1.00	0.00	0.46
G	DPB1*09:01	0.099	1.00	0.06	<0.001
G	DPB1*13:01	0.007	1.00	0.00	0.30
G	DPB1*14:01	0.027	1.00	0.02	0.04
G	DPB1*19:01	0.017	1.00	0.06	0.10

Abbreviation: AIH, autoimmune hepatitis.

### Association between rs9277534 and Clinical Findings

There were no significant differences in the serum levels of albumin, alanine aminotransferase, aspartate aminotransferase, or bilirubin, nor was there one in the frequency of antinuclear antibody positivity, between patients with rs9277534G and rs9277534A.

### Correlation of HLA-DPB1 Expression Level with rs9277534 Genotypes

**Figure 1:**
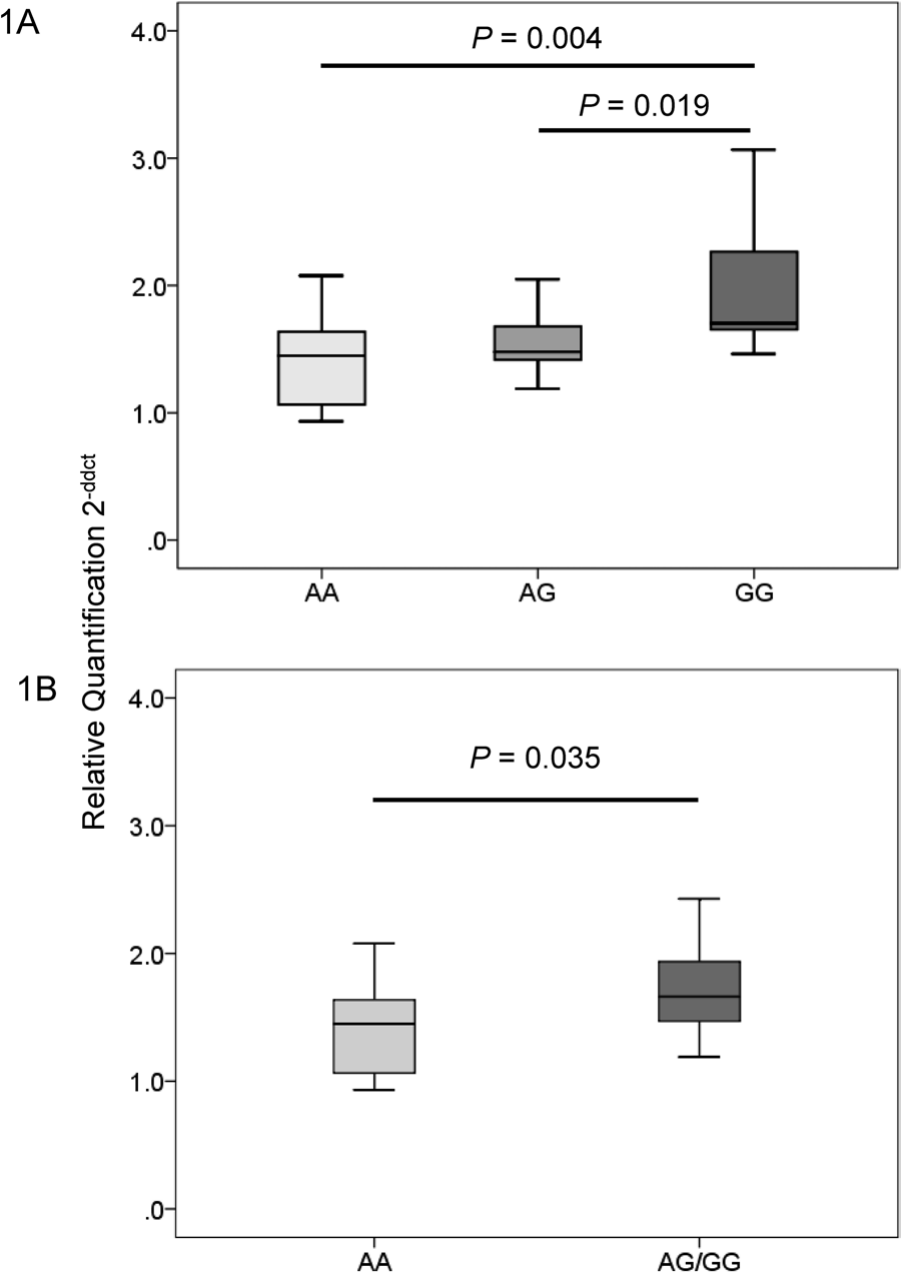
HLA-DPB1 mRNA levels correlate significantly with the rs9277534 allele in the 3′ untranslated region of HLA-DPB1. (**A**) HLA-DPB1 mRNA levels in individuals with rs9277534GG (n = 10) were significantly higher than in those with rs9277534AA (n = 10; *P* = 0.004) or rs9277534AG (n = 10; *P* = 0.019). (**B**) HLA-DPB1 mRNA levels were significantly higher for the rs9277534GG + AG genotypes than for the rs9277534AA genotype (*P* = 0.035).

The rs9277534 genotype reportedly influences the expression level of HLA-DPB1 in European-Americans and African-Americans^21,22^. To validate these findings, the differences in HLA-DPB1 mRNA levels among groups of 10 healthy controls with rs9277534 AA, AG, or GG were determined by quantitative PCR. The median relative quantity of HLA-DPB1 mRNA level was significantly higher in the rs9277534 GG group than in the AA or GA groups (GG: 1.70 vs. AA: 1.45; *P* = 0.004, and vs. AG: 1.48; *P* = 0.019) (Fig. 1A). Moreover, a significant difference was detected for HLA-DPB1 mRNA between the GG + AG and AA genotypes (GG + AG: 1.66 vs. AA: 1.45; *P* = 0.035) (Fig. 1B). Our results confirmed that rs9277534 G-linked HLA-DPB1 mRNA levels were significantly higher than rs9277534 A-linked ones in a Japanese population^21,22^.

## Discussion

In the present study, the rs9277534G allele in the 3′ untranslated region of HLA-DBP1 was strongly associated with AIH compared with healthy subjects in the context of disease susceptibility, although no statistically significant DPB1 allele was detected. Logistic regression analysis showed that rs9277534G was an independent susceptibility allele to AIH in addition to the HLA-DRB1*04:05 allele. rs9277534 is an expression quantitative trait locus (eQTL) that affects gene expression. eQTL SNPs are linked to transcription factor binding, DNA methylation, alternative splicing, small RNA, and other regulatory variants. Therefore, although the functional mechanisms of rs9277434 have not been fully elucidated, this SNP could be indirectly correlated with regulatory elements such as transcription factors.

Our study also confirmed that HLA-DPB1 mRNA expression in cells from Japanese donors correlated with rs9277534A/G genotypes, as reported previously^21,22^. The association of HLA-DP expression with AIH and HBV infection^21^, both of which chronic liver diseases, is intriguing. Although HBV infection is sometimes complicated with autoimmune diseases, its precise mechanisms are unknown^24^. Earlier studies revealed a possible association between AIH and immune T helper (Th)1/Th2 cell balance. Exposure to interleukin (IL)-12 leads to differentiation of Th1 cells secreting interferon-γ, which in turn activates monocytes and cytotoxic CD8 T-cells and promotes natural-killer-cell functions. Interferon-γ also increases major histocompatibility class I and induces major histocompatibility class II expression by hepatocytes, further exacerbating inflammation. IL-4 exposure evokes Th2 differentiation. Th2 cells secrete IL-4, IL-10, and IL-13, all cytokines that enable B-cell maturation into plasma cells with the consequent production of autoantibodies. Ultimately, autoantibodies are involved in antibody-mediated cellular cytotoxicity and complement activation^25^. It is possible that high HLA-DP expression favors a Th2 response characterized by vigorous antibody production, leading to AIH. The titers of anti-liver-specific membrane lipoprotein, asialoglycoprotein receptor, and alcohol dehydrogenase are reportedly correlated with biochemical and histological indices of disease severity^25^. The roles of these autoantibodies in the autoimmune liver attack derive from the finding that hepatocytes are the targets of such immunoglobulins and are susceptible to cytotoxicity. Lastly, whereas Th1 responses are more often observed in HBV recovery, Th2 responses along with poor cytotoxic T-cell activity lead to chronicity^26^. Conversely, lower HLA-DP expression favoring Th1 might be disadvantageous in AIH but effective in HBV recovery. Taken together, higher HLA-DP expression may be deleterious for liver disease in chronic hepatitis B and AIH, while the rs9277534G allele may play an important role in the pathogenesis of chronic hepatitis. Further investigation is needed to clarify this relationship.

The mechanisms underlying the pathogenesis of AIH are not completely elucidated, but several lines of evidence support a strong influence of the large and highly polymorphic HLA region related to adaptive immunity. The most conclusive association with AIH is related to the functional role of HLA class II molecules, particularly HLA-DRB molecules, such as specific allele differences in *DRB1* polymorphisms^5–7,9–11^ and notable amino acid sequence motifs within DRB1 polypeptides^7,10,27,28^. For example, lysine or arginine at position 71, valine or glycine at position 86, and histidine at position 13 reportedly contribute to AIH susceptibility in Northern European Caucasians, Argentina and Brazil, and the Japanese, respectively. Unlike HLA-DR alleles, however, HLA associations with HLA-DP alleles have been insufficiently addressed in AIH, presumably due to a smaller role of DP molecules in immune responses and relatively lower cell surface expression levels^13–15^.

In conclusion, rs9277534G in the HLA-DP gene is an eQTL affecting gene expression that may contribute to AIH susceptibility. Further large-scale studies are required to determine how varied expression of this molecule affects the immune response and development of AIH across ethnicities.

## Methods

### Subjects

Between January 2001 and August 2015, 146 patients with type 1 AIH were enrolled for this study and summarized in Table 5. A total of 326 previously described volunteer healthy subjects were included as well^29^. All controls were hospital staff whose liver function tests were within normal levels and who had indicated the absence of any major illness in a standard questionnaire. The racial background of all individuals was Japanese. All AIH patients had been diagnosed according to the scoring system of the International Autoimmune Hepatitis Group^30^ and were classified as type 1 AIH based on antibody profiles. Anti-nuclear antibody titers were determined by immunofluorescence using HEp-2 cells, in which a titer of ≥1:80 was considered positive^31^. All patients were negative for the hepatitis B surface antigen and antibodies to the hepatitis B core antigen, hepatitis C virus, and human immunodeficiency virus. Other liver diseases and AIH-primary biliary cholangitis overlap syndromes were excluded. All subjects provided written informed consent for this study, which was approved by the Institutional Review Board of Shinshu University Hospital (approval number: 527). The investigation was conducted according to the principals of the Declaration of Helsinki.

**Table 5:** Demographic and Clinical Characteristics of 146 Patients with Type 1 AIH.

Clinical feature		
Age at diagnosis (years)	60	(49–67)
Female, n (%)	138	(89)
AIH score	16	(10–23)
Albumin (4.2–5.1 g/dL)	3.7	(3.3–4.1)
AST (12–37 IU/L)	421	(145–871)
ALT (7–45 IU/L)	426	(155–990)
Bilirubin (0.3–1.2 mg/dL)	1.7	(0.9–6.5)
IgG (870–1700 mg/dL)	2695	(2056–3500)
ANA (< × 40), n (%)	134	(92)

Values are expressed as the median (interquartile range) unless otherwise noted.

Abbreviations: ALT, alanine aminotransferase; ANA, anti-nuclear antibody; AST, aspartate aminotransferase; AIH, autoimmune hepatitis; IgG, immunoglobulin G.

### Genotyping and Linkage Analysis

Genomic DNA from patients and controls was isolated from whole blood samples using QuickGene-800 assays (Fujifilm, Tokyo, Japan). HLA typing in AIH patients was carried out using a Luminex multi-analyzer profiling system with a LAB type SSO One Lambda typing kit (One Lambda, Inc. Canoga Park, CA). HLA genotypes were determined by sequence-based typing, as earlier described^32^. The rs9277534 SNP in the HLA-DP locus was genotyped using a TaqMan 5′ exonuclease assay (Applied Biosystems, Tokyo, Japan). The polymerase chain reaction (PCR) was performed with the StepOne Plus Real-Time PCR System (Applied Biosystems) following the manufacturer’s instructions. Pairwise linkage disequilibrium patterns between HLA-DPB1 alleles and rs9277534 (A/G) were assessed by the Arlequin program (ver. 3.5.2.2) (http://lgb.unige.ch/arlequin/)^33^. The linkage of rs9277534 to *HLA-DPB1* was examined in all AIH patients pursuant to a previous report^23^.

### Measurement of HLA-DPB1 Expression

Blood for RNA analysis was collected using cellular preparation tubes (Becton Dickinson, Franklin Lakes, NJ) from groups of 10 healthy individuals with rs9277534A/A, -A/G, or -G/G genotypes and isolated using a QIAamp RNA Blood Mini Kit (Qiagen, Valencia, CA) according to the manufacturer’s instructions. Single-strand cDNA was synthesized using a high-capacity RNA-to-cDNA kit (Applied Biosystems). Relative quantities of HLA-DPB1 (LifeTechnologies assay ID: Hs00157955_m1) were determined using TaqMan® Gene Expression assays, and the expression of glucuronidase beta (LifeTechnologies assay ID: Hs99999908_m1) was used to normalize gene expression level with the ABI StepOne Plus Real Time PCR system (Applied Biosystems). Real-time data plots were presented as normalized individual data points (2^−deltaCt^ [delta Ct = Ct gene of interest - Ct endogenous control])^34^.

### Statistical analysis

Allele and genotype frequencies along with Hardy-Weinberg equilibrium and linkage disequilibrium were assessed using SNPStats software (Catalan Institute of Oncology, Barcelona, Spain; http://bioinfo.iconcologia.net/SNPstats)^35^ and Haploview 4.1 software^36^. The significance of an association was evaluated using chi-square analysis or Fisher’s exact test. The Mann-Whitney U-test was used to analyze continuous variables. Logistic regression was performed to investigate for associations between the HLA-DRB1*04:05 allele and rs9275534 with susceptibility to AIH. A two-sided *P* value of less than 0.05 was considered to be statistically significant.
